# New-Onset Graves’ Disease With Thyroid Storm After COVID-19 Infection

**DOI:** 10.7759/cureus.47995

**Published:** 2023-10-30

**Authors:** Angela Shermetaro, Jordan Bushman

**Affiliations:** 1 Internal Medicine, Beaumont Hospital, Farmington Hills, Farmington Hills, USA; 2 Endocrinology, Beaumont Hospital, Farmington Hills, Farmington Hills, USA

**Keywords:** graves' disease, thyroid, hyperthyroid, thyroid storm, covid-19

## Abstract

COVID-19 has been a known cause of triggering autoimmune conditions. Previous literature demonstrates an increase in the incidence of Graves’ disease during the COVID-19 pandemic. The virus is thought to affect genetics, leading to a cascade of events that cause hyperthyroidism. In our case, an 81-year-old male presented with symptoms of palpitations, tremors, dizziness, diarrhea, and fatigue. He was found to be in atrial fibrillation with rapid ventricular response, and his workup was consistent with hyperthyroidism. Based on his Burch-Wartofsky score, the diagnosis of thyroid storm was made. There are a limited number of case reports with new-onset Graves’ disease after COVID-19 infection. Interestingly, our patient was also in a thyroid storm. He was treated with hydrocortisone cholestyramine, potassium, and propylthiouracil. After treatment, his symptoms resolved, and his thyroid studies improved. We chose to present this case because it demonstrates one of the many autoimmune effects that COVID-19 has been linked to.

## Introduction

COVID-19 has been a known cause of triggering autoimmune conditions. Previous literature demonstrates an increase in the incidence of Graves’ disease during the COVID-19 pandemic, mainly in women with a history of smoking [1]. The case we present is unique in that our patient was a male with no previously known autoimmune or thyroid conditions that developed Graves’ disease and thyroid storm one week after being diagnosed with COVID-19. There have been a limited number of articles published that suggest COVID-19 as a potential cause of Graves’ disease. Although there is a link between COVID-19 and autoimmune conditions, the mechanism is not completely understood. This article presents a case of Graves’ disease and thyroid storm suspected to be induced by COVID-19.

## Case presentation

An 81-year-old male, with a medical history of hypertension, hyperlipidemia, and benign prostatic hyperplasia, presented to the emergency department for persistent dizziness and weakness. His COVID-19 symptoms were mild as his chest x-ray showed no infiltrate, and he was saturating >94% on room air. He tested positive for COVID-19 one week prior via an at-home test and was started on Paxlovid two days later. Due to confusion with the instructions, he only took 2.5 days' worth of the medication.​​ He then developed tremors, eye irritation, voice hoarseness, headaches, dizziness, and fogginess.​​ He also complained of nausea, watery diarrhea, decreased oral intake, and cough. He reported no family history of endocrine disorders.

On examination, he was in atrial fibrillation with rapid ventricular response with a heart rate in the 140’s. He was admitting to occasional palpitations. He had a fine tremor with outstretched hands. His thyroid was palpable but not enlarged. Workup in the emergency department was significant for a thyroid-stimulating hormone (TSH) level of <0.01 uIU/L (normal: 0.4-4.5 uIU/mL), free triiodothyronine (T3) of 5.3 pg/mL (normal: 1.7-3.7 pg/mL), and free thyroxine (T4) of 2.4 ng/dL (normal: 0.7-1.5 ng/dL). TSH and T4 were within normal range 10 months prior to presentation. The patient had a Burch-Wartofsky score of 45, and the clinical diagnosis of thyroid storm was made.

**Figure 1:**
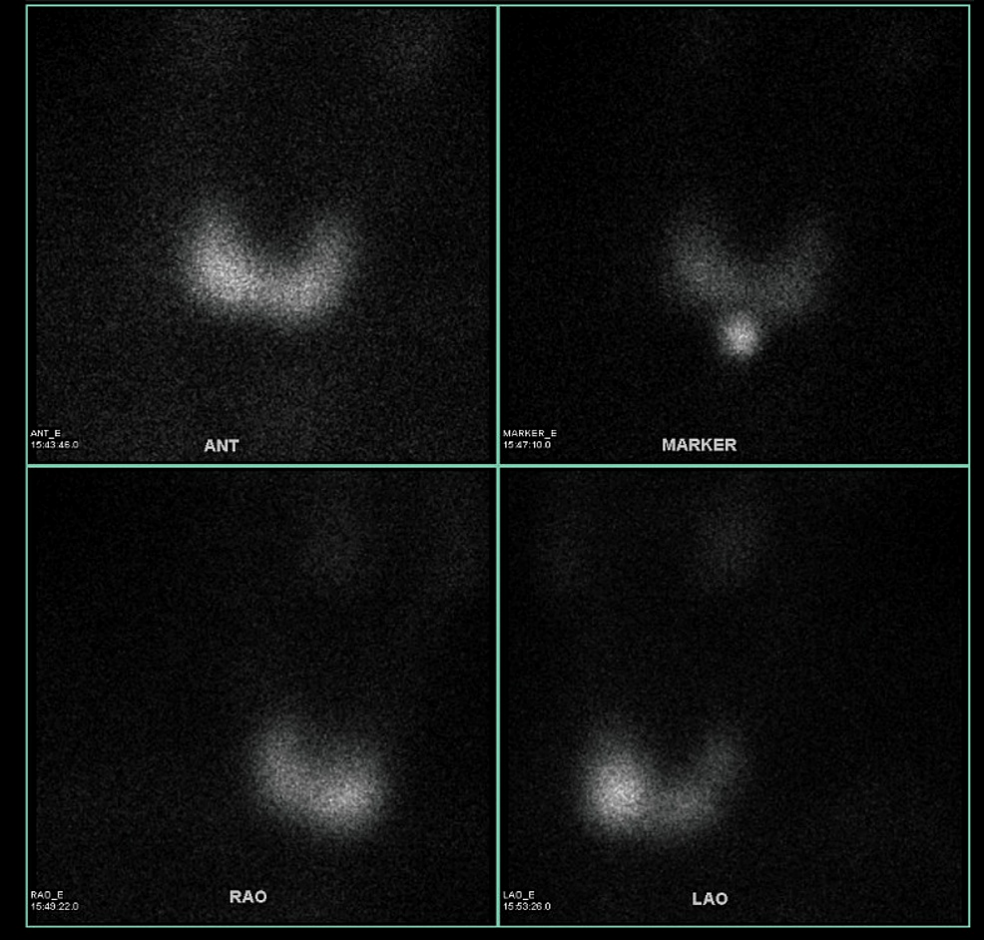
Thyroid Technetium Scan Diffuse increased thyroid activity compatible with hyperthyroidism such as Graves' disease

The following day, his thyroid-stimulating immunoglobulin (TSI) resulted at 12.30 (normal: ​​<0.10 IU/L). His thyroid technetium scan demonstrated homogenous uptake compatible with Graves’ disease (Figure 1).

The patient underwent a CT pulmonary angiogram (CTA PE) given his COVID-19 history, tachycardia, new-onset atrial fibrillation, and a D-dimer of 889 ng/mL. The CTA PE showed no evidence of pulmonary embolism.

The patient was seen by cardiology for new-onset atrial fibrillation with a rapid ventricular response. He was started on an esmolol drip, which was ineffective in controlling his heart rate. He was later loaded with digoxin. He started on metoprolol 25 mg twice daily, and his heart rate was controlled.

Endocrinology started intravenous (IV) hydrocortisone 100 mg every eight hours, cholestyramine 4 g every six hours, potassium iodine 250 mg every four hours, and propylthiouracil 200 mg every four hours. On hospital day three, his free T3 improved to 3.4, and T4 increased to 2.9. He was discharged on methimazole.

Patient followed up with endocrinology, and three months after discharge, his TSH had improved to 3.87. He continues to follow up with our endocrinology clinic, and his methimazole dose has been decreased. He also follows up with cardiology and is on metoprolol for heart rate control.

## Discussion

Graves' disease is an autoimmune thyroid condition, which results from thyrotropin receptor antibodies activating the thyroid receptor and stimulating thyroid hormone synthesis. Researchers do not know what specifically causes people to develop Graves’ disease, but it is thought to be triggered when an outside source such as a virus affects genetics. TSI binds to the TSH receptor and propagates downstream effects of TSH stimulation, leading to hyperthyroidism [2]. The proposed mechanism of COVID-19 viral access to the thyroid is via the angiotensin-converting enzyme 2 (ACE-2) receptor. The ACE-2 receptor is present in the thyroid gland, lungs, kidneys, heart, and adipose tissue [3]. Many mechanisms have been reported to allow the virus to gain access to the thyroid, including the cellular serine protease TMPRSS2 for priming and the αvβ3-integrin, which helps facilitate entry into target organs [4].

We suspect that these mechanisms played a role in our patient's presentation. There have been reported cases of recurrent Graves’ disease after patients have been in remission for over 30 years following COVID-19 infection [5]. However, our patient had no history of Graves’ disease. Cases of new-onset Graves' disease induced by COVID-19 have been reported in the previous literature, including a meta-analysis in clinical endocrinology. However, 97.7% of these subjects were female [1]. A study by Ghareebian et al. describes a case of Graves' disease after COVID-19 in an African-American male with no prior history of thyroid disease [6].

There are multiple autoimmune factors that contribute to Graves' disease, including an increase in the anti-TSH receptor antibody. Our patient also had no previous autoimmune conditions. Interestingly, COVID-19 has been studied as a potential cause of other autoimmune conditions, including HLH, hemolytic anemia, Kawasaki disease, GBS, and Evans syndrome [3].

Our patient presented in thyroid storm based on a Burch-Wartofsky score of 45. This was concluded given his heart rate of greater than 140 beats/minute (25 points), the presence of atrial fibrillation (10 points), and mild central nervous system agitation (10 points). A case of thyroid storm was presented by Sullivan et al. in a 24-year-old female. Sullivan’s article highlights the importance of recognizing the clinical factors of hyperthyroidism, as well as obtaining a thyroid panel, due to the risk of thyrotoxicosis following iodine from a computed tomography angiogram (CTA) study to rule out pulmonary embolism (PE) [7]. This is known as the Jod-Basedow phenomenon, which can occur two to 12 weeks following iodine administration [8]. Our patient did undergo a CTA study due to the high suspicion of PE. He did not have a recurrence of thyrotoxicosis in a two- to 12-week period, as his thyroid studies began to improve at his follow-up visits.

## Conclusions

COVID-19 can trigger Graves' disease in those without any previous autoimmune conditions. The pathophysiology of Graves' disease induced by COVID-19 is not completely understood. Providers must recognize the signs and symptoms of Graves' disease following a COVID-19 infection. Prompt treatment of thyroid storm induced by COVID-19 is important to avoid life-threatening complications.
